# Notes and News

**Published:** 1891-03-14

**Authors:** 


### Notes and News.

Collected and Communicated.

Worcester Infirmary has been suffering from low funds, and there was some talk of drawing on the capital of the institution, when Mr. Lea (of Lea and Perrin's renown) last week generously came forward with a donation of £1,000, and has, as one of the Committee expresses it, "put us on our legs again."

The Bristol General Hospital Committee seem to be in a fair way of realising their wishes in reference to obtaining the £20,000 they are desirous of investing, the interest of which will meet about half of the increased cost that the new wing will entail. The extension scheme provided for a nurses' home, three more hospital wards, a museum, and four small wards for private accommodation. The new wards will not be opened until the £20,000 now asked for has been promised, but as nearly £16,000 has already been contributed it is hoped that the whole amount may soon be obtained.

The fancy fair and bazaar held under the title of "The Coming Race" proved an unqualified success. The opening ceremony was performed by Princess Beatrice, who afterwards received purses containing not less than three guineas, at the hands of several ladies and children. The West-end Hospital, in aid of which the fête was organised, is the largest devoted to the treatment of nervous complaints, the number of attendances in the course of last year being nearly 28,000. It has been considerably enlarged at great expense, and funds are needed to enable the managers to admit more patients. It is also desired to found scholarships and exhibitions, to enable ladies to be trained in the school of massage and electricity associated with the hospital.

The nineteenth annual meeting of the Governors of the New Hospital for Women was held at the hospital on the 4th inst., the Rev. T. Llewelyn Davies in the chair. The report showed that the number of in-patients treated was 213, and the number of out-patients 5,775. The balance-sheet showed that the expenses were £2 263, and the total receipts were £2,380, leaving a balance of £127. To the building fund £20,000 had been added from various sources, but chiefly from the profits of a bazaar which was held in the new building at the end of April, which produced a clear profit of £1,721. The number of beds has been increased in the hospital from 26 to 42. The report was adopted and the proceedings closed.

The report of the Committee on Hospital Reform at Birmingham is practically to the effect that there has been a great deal of smoke to very little fire, and that our hospitals are not much abused. In conclusion, the Committee recommend: 1. The formation of a General Council representative of all the public medical institutions of the city. 2. The formation of an Inquiry Agency to investigate the circumstances of applicants for treatment at the hospitals. 3. That apart from first aid and urgent cases, regulations should be framed by the hospitals to exclude trivial cases, and cases where either the patients are in a position to pay for such treatment as they may require, or which could be more properly dealt with under the Poor-law. 4. That facilities should be given for cases so excluded being dealt with by dispensaries or provident associations. 5. That any person recommended by an approved provident association or by a qualified medical practitioner, should, as a rule, be admitted to the out-patient departments of the hospitals without further formality.

The good work of that excellent institution, the Hospital of St. John of Jerusalem and St. Elizabeth of Hungary, continues to grow and prosper. The fifty beds it contains were all occupied during the whole of last year, and the many cases which had to be refused or deferred through want of accommodation, have made the enlarging and re-building of the hospital an immediate necessity. It will probably require some £10,000 to carry this work out efficiently, towards which £1,400 has already been subscribed. This hospital, which was established for the reception of women suffering from incurable or long-standing diseases, appeals for support on two special pleas namely, that it depends wholly on voluntary contributions and that its whole administration, nursing, and medical attendance, are gratuitous.

Mr. G. P. Wyatt held a lengthened inquiry at St. Thomas's Hospital touching the death of George William Nicholas, aged 22, who was fatally injured in one of the hydraulic lifts in the hospital. The deceased was a compositor, and had been under treatment in the hospital for an affection of the spine since September last. It was proposed to move him elsewhere for further treatment. The deceased was placed on a bed, and, this being put on a trolley, was wheeled from the Edward Ward to the lift. A police-constable named Seppings, at the instance of one of the porters, held the rod by which the lift is worked to keep the apparatus steady. Suddenly, however, just as the trolley was being moved on to it, the lift flew up, and the deceased and the bed became wedged between the edge of the lift and the wall. The man on being extricated, died a few moments afterwards from the shock, having, singularly enough, according to the medical evidence, escaped outward injury. There was no evidence to show how the lift came to move. The jury returned a verdict of "Accidental death," and added that the lift should be better lighted.

A hospital which has a very strong claim upon the merchants and citizens of London is the Royal Hospital for Children and Women, Waterloo Bridge Road, it having been in the first instance founded in, and being particularly an institution of, the City of London. During the past year (1890) 509 in-patients have been treated, against 354 in 1889; 6,710 out-patients have made 27,154 attendances, against 7,105 making 29,586 in the previous year; and there have been 310 dental cases, as compared with 314. Altogether, since the hospital was started through the exertions of Dr. J. Bunnell Davis in 1816, 750,000 patients have been relieved. The income of the hospital has amounted to £3,550, against £2,946 in the previous year, and of this amount £300 has been added to the Extension Fund (which now amounts to £400), and £470, being legacies, have been invested in Consols. At the annual general Court of Governors, held on the 6th inst. at the Mansion House, the Lord Mayor (who was accompanied by the Lady Mayoress) presiding, supported by Mr. Sheriff Harris, Mr. Sheriff Farmer, and a number of other ladies and gentlemen, friends of the hospital, a subscription list was opened to make up the annual deficit, and something like £300 was subscribed in the room, about £100 more than the annual meeting generally realises.

The Lord Mayor said the annual meeting was usually held at the hospital, but he considered that if any meetings were held at the Mansion House, they always excited more enthusiasm than if held at the institutions. This institution was worthy of the earnest support, and as it was worked on most economical principles, he made an earnest appeal for funds. We commend this hospital to public sympathy, and hope many people will send a contribution to the able secretary, Mr. Kestin.

